# Exploring Long-Term Neurocognitive Impact of Pineal Region Tumors: Clinical and Therapeutical Perspectives from a Single-Center Study

**DOI:** 10.3390/children13020197

**Published:** 2026-01-30

**Authors:** Flavio Panico, Antonio Verrico, Maria Sole Venanzi, Maria Grazia Calevo, Diego Garbossa, Claudia Milanaccio, Gianluca Piatelli, Alessandro Consales

**Affiliations:** 1 Neurosurgery Unit, Department of Neuroscience “Rita Levi Montalcini”, A.O.U. Città Della Salute e Della Scienza Torino University of Turin, 10124 Turin, Italy; 2 Neurooncology Unit, IRCCS Istituto Giannina Gaslini, 16147 Genoa, Italy; 3 Department of Neurosurgery, IRCCS Istituto Giannina Gaslini, 16147 Genoa, Italy; 4 Epidemiology and Biostatistics Unit, IRCCS Istituto Giannina Gaslini, 16147 Genoa, Italy

**Keywords:** pineal tumors, germ cell tumors, pediatric tumors, neurocognitive outcome, pineal gland, melatonin

## Abstract

*Purpose:* Pineal region tumors are rare tumors in the pediatric population, typically managed with surgical resection or biopsy, and often with radiation therapy and chemotherapy. This study aims to examine the clinical and neurocognitive outcomes of pediatric patients with pineal tumors. *Methods:* A retrospective analysis was conducted on pediatric patients with pineal region tumors treated at Istituto Giannina Gaslini, Genoa, from January 1998 to July 2023. Data on medical history, surgical approaches, histological findings, administered therapies, long-term outcomes using the Glasgow Outcome Scale (GOS), education level, and employment status were collected. Statistical analysis was performed using SPSS software. *Results:* We identified 38 patients, with germinoma being the most prevalent tumor (47.4%). Surgical interventions included endoscopic biopsy (20 patients), stereotactic biopsy (5 patients), and excisional surgery (5 patients). Thirty-three patients received chemotherapy, and 35 underwent adjuvant radiotherapy. The mean follow-up duration was 8.79 ± 5.71 years. Significant correlations were found between tumor dissemination at diagnosis and patient outcomes (*p*-value = 0.03). Notably, patients in GOS classes 5–6 did not significantly differ from those in classes 7–8 regarding the frequency of intervention. School dropout rates significantly differed between GOS classes 5–6 and 7–8. *Conclusions:* This study highlights that prognosis is strongly associated with tumor aggressiveness, and particularly dissemination at diagnosis. The findings also suggest potential cognitive impairments, possibly linked to melatonin dysfunction induced by tumor-related treatments, as indicated by school dropout and employment data. *Implications for Cancer Survivors*: Our results underscore the need for further investigation into the impact of pineal involvement and potential therapeutic strategies.

## 1. Introduction and Purpose of the Study

Pineal region tumors constitute a significant yet relatively minor subset, accounting for approximately 2.8% of pediatric brain tumors and 1% of primary central nervous system tumors, thus posing a distinct challenge for clinicians and neurosurgeons. This tumoral category encompasses neoplasms originating from pinealocytes, germ cells, and neuroectodermal tissue [1,2].

The nature of these tumors, coupled with their deep-seated anatomical location and proximity to critical structures [3], often necessitates a multifaceted therapeutic approach. In some cases, surgical intervention is required to address the associated non-communicating hydrocephalus.

The comprehensive management of malignant pineal tumors, indeed, typically involves surgical resection or biopsy, complemented by radiation therapy and chemotherapy. Biopsy options, including stereotactic, endoscopic, and open microsurgical approaches, play a pivotal role. The neuroendoscopic approach often emerges as a strategic choice, providing dual benefits of obtaining a histological diagnosis and concurrently addressing non-communicating hydrocephalus [4,5].

For symptomatic low-grade tumors such as pineocytomas, pineal cysts, pilocytic astrocytoma, and mature teratoma, surgical resection represents the definitive treatment. For other malignant lesions, surgery is integrated with radiotherapy and chemotherapy [6].

However, when a pineal region tumor presents with elevated levels of alpha-fetoprotein (AFP) and beta-human chorionic gonadotropin (β-HCG) in either serum or cerebrospinal fluid, additional histological confirmation is deemed unnecessary [7]. Indeed, according to European guidelines endorsed by the International Society of Pediatric Oncology (SIOP), a serum and/or CSF AFP level equal to or higher than 25 ng/mL and/or β-HCG level equal to or higher than 50 IU/L defines the presence of a secreting non-germinoma germ cell tumor (NGGCT) [8].

This retrospective study aims to share our experiences, delving into patients’ clinical features, therapeutic strategies (both surgical and non-surgical), and the subsequent clinical outcomes, with particular emphasis on neurocognitive function. Additionally, we analyze factors such as educational background and employment status to explore their potential impact on patient outcomes.

## 2. Materials and Methods

Following Ethical Committee approval, a retrospective analysis was conducted on data collected from a single-center database regarding pediatric patients with a diagnosis of pineal region tumor treated at IRCSS Istituto Giannina Gaslini, Genoa, from January 1998 to July 2023. Eligible patients were between 1 and 18 years old with a follow-up of at least 6 months.

We reviewed hospital discharge summaries, follow-up visits, operative, radiological, and anatomical pathology reports. Data on medical history, surgical approaches, histological findings, and administered chemo- and radiotherapy were extracted for the analysis. 

Furthermore, a prospective long-term outcome analysis was conducted utilizing the extended form of the Glasgow Outcome Scale Questionnaire (GOSE), which was administered by trained clinicians through structured interviews at the last available follow-up. Patients were categorized into three GOSE groups: GOSE 1–3, GOSE 5–6, and GOSE 7–8.

Outcome assessment encompassed two additional key parameters for a more comprehensive evaluation: patients’ level of education and employment status. Patients were stratified based on their level of education as high, medium, or low. Patients were divided into patients with high schooling (who achieved at least a university degree at least), medium schooling (high school diploma), and low schooling (middle school). To better capture the real-life functional impact of disease and treatment and to mitigate potential age-related bias, information regarding school dropout status—defined as failure to achieve an educational level appropriate for the patient’s age, regardless of the final degree obtained—was also collected. Next, patients were categorized according to their engagement in work activities as “engaged” or “not engaged”. Additionally, the presence or absence of employment difficulties was noted. Educational attainment, school dropout, and employment status were used as functional real-life outcome measures to reflect long-term everyday functioning rather than specific neurocognitive domains.

The data underwent descriptive analysis, with frequencies and percentages calculated for qualitative variables, while continuous variables were characterized using the mean, standard deviation, median, and range. Stratification of study data was performed based on age group, gender, histologic type, and tumor stage (diffuse/localized).

The normality of variables was assessed using the Kolmogorov–Smirnov test. Depending on the obtained result, a parametric or nonparametric statistical approach was used. Continuous variable group comparisons utilized ANOVA or Kruskal–Wallis tests as well as Mann–Whitney’s U tests, while associations between categorical variables were examined using the χ2 test or Fisher’s exact test.

All *p*-values were calculated using two-tailed tests, with statistical significance defined as a *p* < 0.05. Statistical analyses were conducted using SPSS version number 18 for Windows (SPSS Inc, Chicago, IL, USA).

### Diagnostic and Therapeutic Work-Up

All patients underwent a thorough diagnostic evaluation, encompassing the assessment of tumor markers, and advanced imaging modalities, such as computed tomography (CT) scans and magnetic resonance imaging (MRI) (including T1-weighted imaging, T2-weighted imaging, gadolinium-enhanced T1 with fat suppression, fluid-attenuated inversion recovery, and diffusion-weighted imaging). However, data on tumor markers were not available for patients who arrived from secondary centers.

A multidisciplinary neuro-oncology board tailored a comprehensive surgical and clinical management plan guided by imaging, hydrocephalus, tumor marker presence, and surgeon preferences.

The selection of an appropriate surgical approach depends on factors such as tumor size, its location relative to the Galen venous complex, and the surgeon’s proficiency [9]. Over the past two decades, various surgical techniques, including occipital transtentorial (OTT) [10], infratentorial supracerebellar (SCIT) [11], and interhemispheric transcallosal (ITC) [12] approaches, have been extensively investigated. The OTT approach, performed with the patient seated or in a three-quarter prone position (park bench), is preferred for tumors extending upward with displacement of the venous complex downward, providing direct access to the pineal region. Conversely, the SCIT approach, conducted with the patient in a seated position, is favored for tumors extending posteriorly, facilitating a direct surgical pathway. Therefore, these three surgical approaches are the most commonly utilized for the removal of pineal lesions, with careful consideration given to individual patient circumstances. These kinds of surgical approaches have already been extensively reviewed in the literature and are known to be the surgical techniques of choice for this type of pathology, thus should be carefully selected based on surgeon preference and individual circumstances. Furthermore, our main objective of this study was within the analysis of the long-term outcomes that these pathologies determine. For these reasons, they were not considered in our statistical analyses.

Management strategies for patients with concurrent acute hydrocephalus varied with options ranging from external ventricular drainage (EVD), endoscopic third ventriculostomy (ETV), and ventriculoperitoneal shunting (VPS) to direct surgical excision [13]. Conversely, in cases of slow progressive hydrocephalus, direct surgical removal of the lesion was typically favored.

## 3. Results

### 3.1. Study Population, Diagnostic and Therapeutical Management, and Clinical Follow-Up

Thirty-eight patients were included in the study, comprising 32 males and 6 females, with a mean age at diagnosis of 12.11 ± 5.53 years.

In 13 patients, clinical and radiological findings led to a diagnostic conclusion without necessitating biopsy due to either bifocal or disseminated lesions with neuroimaging evocative of germinoma, or elevated levels of alpha-fetoprotein and/or beta-human chorionic gonadotropin found in cerebrospinal fluid/serum. Diagnoses included 6 pinealoblastomas, 18 germinomas (7 bifocal and 4 disseminated), 10 non-germinomatous germ cell tumors (NGGCT, 6 disseminated), 3 teratomas (1 immature and 2 disseminated), and 1 pilocytic astrocytoma. The most common tumor entity was Germinoma (47.4%) according to the pediatric population of our series.

The diagnostic-therapeutic protocol involved biopsy collection and simultaneous management of hydrocephalus, if present. Twenty patients underwent biopsy sampling of the lesion only (excision less than 10% of the lesion). Of these, 15 patients benefited from endoscopic biopsy sampling at the same time as ETV surgery. In one patient, biopsy sampling was not diriment to histologic analysis. Five patients, on the other hand, underwent stereotactic biopsy sampling; again, only one biopsy sampling was not diriment. Three patients underwent excisional surgery obtaining a subtotal resection (nodular residue greater than 50% at postoperative magnetic resonance imaging), another three underwent gross total resection (no lesional residue at postoperative MRI check) while in two patients, a partial excision was obtained (lesional residue at postoperative MRI check between 10 and 50% of the lesion). Among the 38 patients, 29 (76.32%) exhibited hydrocephalus. Initial treatment for hydrocephalus consisted of ETV in 22 cases, while 5 patients underwent EVD. Among the latter group, two patients subsequently required ETV, one patient underwent internalization of the shunt via VPS. Lesion debulking surgery was successful in the remaining two cases affected by obstructive hydrocephalus.

Additionally, apart from the patient who underwent EVD internalization, VPS placement was subsequently performed in eight other patients following biopsy, bringing the total to nine patients who underwent VPS.

On average, patients underwent approximately 1.23 ± 1.16 tumor excision surgeries each. However, for those presenting with hydrocephalus, the average number of surgeries per patient increased to 1.46 ± 1.29, inclusive of procedures such as VPS or ETV surgeries. Consequently, each patient underwent a total of 2.23 ± 2.06 surgeries.

Elevation of serum and CSF levels of β-HCG and AFP were also analyzed Among the 38 patients, 22 (57.89%) did not show any abnormalities in either marker. A diagnosis of NGGCT was supported by above-average levels of both AFP and β-HCG in both CSF and serum in two patients (5.26%), of AFP levels both CSF and serum in three patients (7.89%), of β-HCG levels both CSF and serum in four patients (10.52%), and of only CSF β-HCG levels in one patient. The analysis of CSF and serum markers in six patients (15.79%) could not be determined.

Regarding chemotherapy, only 5 out of 38 patients (13.16%) did not receive chemotherapy. Among those who did, 26 patients were treated according to the SIOP CNS GCTs 96 scheme [14], 3 patients were treated according to the AIEOP Medulloblastoma and Primitive Neuroectodermal Tumor high-risk scheme [15] for over 3 years, and another 3 patients were treated with the SNC HR protocol [16] for less than 3 years. In only one patient was the administered chemotherapy treatment impossible to determine. Additionally, nine patients underwent high-dose chemotherapy treatment. On average, each patient underwent 1.17 ± 0.75 lines of chemotherapy.

Thirty-five patients (92.11%) received radiation treatment, while only three patients (7.89%) did not. Specifically, 15 out of 35 patients (42.86%) underwent craniospinal irradiation (CSI), while 20 patients (57.15%) received focal radiotherapy.

The mean follow-up duration ± standard deviation (SD) was 8.79 ± 5.71 years (ranging from 6 months to 19 years).

Following surgical, radiotherapy, and chemotherapy interventions, post-treatment sequalae were observed. Specifically, campimetric deficits were noted in 5 patients, ocular motility issues in 3 patients, and endocrine-metabolic complications, including 10 cases of panhypopituitarism, were reported in 12 patients. Additionally, two patients exhibited depressive symptoms, and one patient was diagnosed with attention deficit hyperactivity disorder (ADHD).

### 3.2. Clinical Outcome Based on GOSE, Schooling, and Employment Status

In the methodology outlined in this study, patients were stratified into three distinct groups based on outcome assessment parameters.

We administered the GOSE questionnaire to 35 patients out of a total of 38.

Within the first group (GOSE classes 1–3), there were three patients (three patients GOSE class 1); in the second group (GOSE classes 5–6) there were nine patients (one patient in GOSE class 5 and eight patients in GOSE class 6); and finally in the third group, there were (GOSE classes 7–8) twenty-four patients (twenty-three patients in GOSE class 7).

Notably, a statistically significant correlation (*p*-value = 0.03) existed between the extent of dissemination at diagnosis and patient outcomes. All deceased patients (4/4 GOSE 1) had dissemination detected at diagnosis. Moreover, patients classified within GOSE classes 5–6 did not significantly differ from those in GOSE classes 7–8 in terms of the number of interventions; nevertheless, they exhibited distinct outcome trajectories.

Notably, we observed a statistically significant difference between the presence of dissemination at diagnosis and patient outcomes (*p*-value = 0.03) (Table 1). All deceased patients (3/3 (100%) GOSE 1) had dissemination detected at diagnosis versus 44.4% of GOSE 5–6 patients and 26.1% of GOSE 7–8 patients. Moreover, patients classified within the GOSE classes 5–6 did not significantly differ from those in GOSE classes 7–8 in terms of the total number of interventions (2.67 ± 2.55 vs. 1.96 ± 1.92 *p* = 0.43); nevertheless, they exhibited distinct outcome trajectories. Patients with intermediate GOSE scores (GOSE 5–6) required VPS in a larger proportion of cases with respect to patients in GOSE 7–8 (55.6% vs. 13%; *p* = 0.02) (Table 1).

Concerning the employment status in alive patients with age ≥ 18 years, nine patients were employed without any statistically significant difference between GOSE (2 (40%) vs. seven (41.2%); *p* = 1) (Table 1).

Abnormal school dropout was observed in 15 patients. Statistical significance was observed in school dropout rates between GOSE classes 5–6 (7 out of 9 patients, 77.8%) and GOSE classes 7–8 (8 out of 23 patients, 34.8%) (*p* = 0.05) (Table 1).

## 4. Discussion

In the literature, pineal region tumors represent a small fraction of all central nervous system (CNS) tumors, accounting for 0.5% in adults, 1% in young adults (aged 20–34 years), and 2.7% in children (aged 1–12 years) [17]. Among these, germ cell tumors (GCTs) are notably prevalent, constituting the most commonly encountered group with incidence rates ranging from 50% to 75% [18]. This study offers insights from our institution’s management of 38 cases of pineal region tumors. Noteworthy within our cohort is the high prevalence of GCTs, with an incidence rate of 81.6%, exceeding figures reported in larger databases such as the SEER database (58.9%) [19] and the French National Register (27%) [20]. Germinoma emerged as the predominant histological subtype, representing 47.4% of cases, consistent with data from the Japanese registry [21]. Additionally, a male predominance of 75% was observed, aligning with findings documented in the U.S. registry. This histological distribution, although consistent with institutional referral patterns, contributes to cohort heterogeneity and should be considered when interpreting outcome data. In particular, different histological subtypes are associated with distinct biological behavior, treatment intensity, and risk of dissemination, all of which may variably influence the long-term functional and neurocognitive outcomes. Consequently, the observed outcomes likely reflect the combined effect of tumor biology and overall treatment burden rather than histology alone.

In our experience, when encountering cases of obstructive triventricular hydrocephalus, we prefer to perform an ETV (and an eventual associated endoscopic biopsy), as reported in the literature [22], rather than opting for an EVD. This preference is based on the fact that EVD is typically a temporary measure, subject to subsequent internalization or removal. Consequently, patients often require further intervention, as evidenced by our observation that out of five patients who underwent EVD, two necessitated additional ETV procedures.

As expected, the quod vitam prognosis is evidently linked to the aggressiveness level of the lesion [23], as demonstrated by the statistically significant correlation between the extent of dissemination at diagnosis and patient outcomes (*p* = 0.03). Elevation of blood or CSF markers such as B-HCG or aFP is known to be pathognomonic for the presence of malignant germ cell elements [24] and was reported in 6 out of 15 patients with dissemination at diagnosis out of a total of 10 patients with alteration of these markers.

In our analyzed case series, looking at the total number of surgeries (for hydrocephalus and for oncology purposes), patients underwent an average of 2.7 surgeries for those in GOSE classes 5–6 and 1.9 for those in GOSE classes 7–8, revealing a minimal discrepancy in intervention frequency across different GOSE classes. A higher proportion of patients in GOSE classes 5–6 required VPS procedures (55.6% vs. 13% in GOSE 7–8), with all the eventual associated complications [25]. This could be attributed to dissemination at diagnosis. Notably, patients with a disseminated tumor at diagnosis often necessitate craniospinal radiotherapy, which can lead to poorer neurocognitive outcomes compared to limited loco-regional treatment, as evidenced in long-term studies [23,26,27,28,29,30]. The impact of radiotherapy dose and fields on neurocognitive outcomes is well-established in the literature; therefore, in this small and heterogeneous cohort, radiotherapy was considered as part of the overall treatment burden rather than analyzed as an independent determinant of cognitive outcome.

In the context of neurocognitive outcomes, an important aspect worthy of discussion is school dropout rate. Specifically, if dissemination at diagnosis emerges as an independent risk factor for school dropout, it is intriguing to observe that among the 15 patients who dropped out of school, 8 were classified as GOSE 7–8. This finding highlights the potential dissociation between global functional outcome scales and more subtle long-term educational or cognitive difficulties. A hypothesis that could be posited in light of these findings, considering the extended follow-up period (mean duration: 8.79 ± 5.71 years, with only 3 patients having less than 1 year of follow-up and 28 with at least 4 years of follow-up), is that pineal involvement might lead to impairments in higher cognitive functions independent of disease extent and treatment modalities. This hypothesis finds support in the employment data: 4 out of 30 patients (for whom employment data were available) experienced difficulties in finding employment, and all of them dropped out of school. Among these individuals, 2 exhibited normal GOSE scores, while 2 reported relationship issues and depressive symptoms. These observations suggest that real-life functional outcomes may capture aspects of long-term neurocognitive and psychosocial impact that are not fully reflected by standard outcome scales alone. A possible explanation is linked to melatonin dysfunction resulting from damage to the glandular parenchyma induced by radiotherapy, surgery, or the tumor itself [31]. Recent studies have revealed that a reduced melatonin production seems to correlate with neurodegenerative and psychiatric disorders [32]. Furthermore, in vitro studies have demonstrated melatonin’s significant protective effects against oxidative damage to brain tissue [33]. However, in the absence of direct melatonin measurements in our cohort, this proposed mechanism remains speculative and should be interpreted as hypothesis-generating, underscoring the need for future prospective studies specifically addressing pineal function and neurocognitive outcomes.

## 5. Limitations

The main limitations of this study include the retrospective single-center design and the limited sample size, which reflect the rarity of pediatric pineal region tumors and reduce the statistical power to detect subtle associations. In particular, the GOSE 1–3 subgroup included only three patients; therefore, comparisons involving this group must be interpreted with caution and are mainly descriptive rather than inferential. Moreover, our cohort included heterogeneous histologies and treatment regimens over a long inclusion period, potentially confounding outcome interpretation and limiting the possibility of attributing functional outcomes to a single clinical or therapeutic factor. With respect to neurocognitive outcomes, we acknowledge that formal standardized neuropsychological testing was not consistently available in this long-term retrospective series. Therefore, we intentionally relied on functional and real-life outcome measures such as educational attainment/school dropout, and employment status to capture the practical impact of the disease and its treatments on patients’ everyday functioning and social integration. While these measures do not assess specific neurocognitive domains, they provide meaningful information on long-term functional consequences in real-world settings. Finally, although we discuss a potential link between pineal involvement, melatonin dysfunction, and higher cognitive impairment, this remains hypothesis-generating because melatonin measurements were not available in our cohort. Similarly, detailed radiotherapy dosimetry (dose and fields) and some treatment details were not uniformly retrievable across the entire study period, further limiting treatment-specific conclusions.

## 6. Conclusions

The presented data constitute a series of pediatric patients with different subtypes of pineal region tumors. Our findings suggest that long-term functional outcomes, including school dropout rates, may be affected by pineal region tumors, even in patients with favorable global outcome scores, highlighting the potential discrepancy between standard outcome scales and real-life functioning. The long-term follow-up indicates a possible impact of pineal tumors on higher cognitive and psychosocial functioning. The hypothesized role of melatonin disruption should be considered exploratory and hypothesis-generating, as no direct measurements were available. These observations should be interpreted in light of the retrospective design, limited sample size, and cohort heterogeneity.

Overall, our study highlights the multifaceted nature of pineal region tumors and emphasizes the importance of comprehensive, long-term functional assessment in pediatric survivors. Further research is needed to validate our findings and explore novel therapeutic avenues aimed at mitigating neurocognitive sequelae associated with these challenging tumors.

## Figures and Tables

**Table 1:** Demographic and clinical characteristics stratified by GOSE scores.

	GOSE			
	GOSE 1	GOSE 5–6	GOSE 7–8	*p*-Value
	N = 3	N = 9	N = 23	
	N (%)	N (%)	N (%)	
**Gender, M**	3/3 (100)	8/9 (88.9)	20/23 (87)	0.67
**Dissemination detected at diagnosis, yes**	3/3 (100)	4/9 (44.4)	6/23 (26.1)	**0.03**
**Radiotherapy, yes**	3/3 (100)	8/9 (88.9)	21/23 (91.3)	0.74
**CT, yes**	3/3 (100)	7/9 (77.8)	21/23 (91.3)	0.42
**HDCT, yes**	2/3 (66.7)	2/9 (22.2)	4/23 (17.4)	0.22
**Hydrocephalus, yes**	3/3 (100)	8/9 (88.9)	18/23 (78.3)	0.43
**VPS, yes**	2/3 (50)	5/9 (55.6)	3/23 (13)	**0.02**
**Tumor exeresis, yes**	3/3 (100)	7/9 (77.8)	17/23 (73.9)	0.43
**Death, yes**	3/3 (100)	0/9 (0)	0/23 (0)	**0.0001**
		N = 9	N = 23	
**School dropout, yes**	Not applicable	7/9 (77.8)	8/23 (34.8)	**0.05**
Worker, yes *	Not applicable	2/9 (40)	7/23 (41.2)	1

M—male, CT—chemotherapy, HDCT—high-dose chemotherapy, VPS—ventriculoperitoneal shunt. * Patients alive with age ≥ 18 yrs (N = 22).

## Data Availability

The data presented in this study are available on request from the corresponding author. The data are not publicly available due to privacy and ethical reasons.
